# A soft X-ray free-electron laser beamline at SACLA: the light source, photon beamline and experimental station

**DOI:** 10.1107/S1600577517015685

**Published:** 2018-01-01

**Authors:** Shigeki Owada, Kazuaki Togawa, Takahiro Inagaki, Toru Hara, Takashi Tanaka, Yasumasa Joti, Takahisa Koyama, Kyo Nakajima, Haruhiko Ohashi, Yasunori Senba, Tadashi Togashi, Kensuke Tono, Mitsuhiro Yamaga, Hirokatsu Yumoto, Makina Yabashi, Hitoshi Tanaka, Tetsuya Ishikawa

**Affiliations:** a RIKEN SPring-8 Center, Sayo-cho, Sayo-gun 679-5148, Japan; b Japan Synchrotron Radiation Research Institute, Sayo-cho, Sayo-gun 679-5948, Japan

**Keywords:** free-electron laser, soft X-ray, beamline instrumentation

## Abstract

The design and performance of a soft X-ray free-electron laser (FEL) beamline of the SPring-8 Compact free-electron LAser (SACLA) are described. The SPring-8 Compact SASE Source test accelerator, a prototype machine of SACLA, was relocated to the SACLA undulator hall for dedicated use for the soft X-ray FEL beamline. Since the accelerator is operated independently of the SACLA main linac that drives the two hard X-ray beamlines, it is possible to produce both soft and hard X-ray FEL simultaneously. The FEL pulse energy reached 110 µJ at a wavelength of 12.4 nm (*i.e.* photon energy of 100 eV) with an electron beam energy of 780 MeV.

## Abstract 2

The status of a soft X-ray free-electron laser beamline at SACLA is reported.

## 1. Introduction   

The advent of X-ray free-electron lasers (XFELs) has stimulated marked progress in various scientific fields, such as ultrafast chemistry (Moshammer *et al.*, 2007 ▸; Erk *et al.*, 2014 ▸), nonlinear X-ray optics (Rohringer *et al.*, 2012 ▸; Glover *et al.*, 2012 ▸; Bencivenga *et al.*, 2015 ▸) and structural biology (Boutet *et al.*, 2012 ▸; Nango *et al.*, 2016 ▸). The first FEL operation in the extreme ultraviolet (EUV) region was achieved by the TESLA Test Facility (TTF; Andruszkow *et al.*, 2000 ▸) at DESY, Germany, in 2000, which was later renamed FLASH (Ackermann *et al.*, 2007 ▸). The FEL wavelengths were shortened to the angstrom region by the Linac Coherent Light Source (LCLS) (Emma *et al.*, 2010 ▸) at the SLAC National Accelerator Laboratory (SLAC), USA, in 2009. A soft X-ray FEL based on a high-harmonic high-gain (HGHG) seeding scheme was also in operation at FERMI in Elettra, Italy (Allaria *et al.*, 2012 ▸).

The SPring-8 Ångstrom Compact free-electron LAser (SACLA), constructed in Harima, Japan, achieved first lasing at 10 keV in June 2011 and started operation for users in March 2012 with two beamlines: BL3 for a hard X-ray FEL, which is capable of generating the shortest wavelength FEL below 0.8 Å, and BL1 for wide range spontaneous emission (Ishikawa *et al.*, 2012 ▸; Yabashi *et al.*, 2015 ▸). Based on unique capabilities and continuous upgrades (Hara *et al.*, 2013 ▸; Mimura *et al.*, 2014 ▸; Katayama *et al.*, 2016 ▸), BL3 has been providing research opportunities in various fields of science (Shwartz *et al.*, 2014 ▸; Tamasaku *et al.*, 2014 ▸; Yoneda *et al.*, 2014 ▸, 2015 ▸; Kim *et al.*, 2015 ▸; Inoue *et al.*, 2016 ▸). The success of BL3 and users’ demands for higher availability led us to construct the second XFEL beamline (BL2), which has been operating since April 2015. At the same time, a much smaller number of research proposals applied for BL1, mainly because only spontaneous radiation was available with one undulator unit of 4.5 m length. In addition, the operation of BL1 limited the availability of BL2 and BL3, because the SACLA main linac had been commonly employed to all beamlines.

However, requirements for utilizing soft X-ray FELs have been continuously increasing. To meet the demands, we commenced a project to upgrade BL1 in 2014. A marked feature was to re-employ the SCSS test accelerator (SCSS; Shintake *et al.*, 2008 ▸) as an electron beam driver dedicated to BL1, independent of the SACLA main linac operating for BL2 and BL3; the SCSS was relocated to a 90 m-long empty space on the upstream side of the dog-leg section of BL1 in the SACLA undulator hall, which can accommodate high-gradient C-band accelerator units to generate a several hundred MeV to ∼1 GeV electron beam.

The original SCSS machine was constructed as a prototype of SACLA for evaluation of the feasibility of a compact XFEL concept as well as development of experimental techniques with FELs. The construction of the SCSS with a 250 MeV linac and two undulators was completed in 2005. The first lasing at a wavelength of *λ* = 49 nm was achieved in 2006, followed by user operation in 2007 (Sato *et al.*, 2008 ▸). Furthermore, the first high-harmonic-generation (HHG) seeded FELs in the ultraviolet (UV) and EUV region were demonstrated (Lambert *et al.*, 2008 ▸; Togashi *et al.*, 2011 ▸; Tomizawa *et al.*, 2015 ▸). After these achievements and successful operation of SACLA, the SCSS was decommissioned in 2013.

In 2014, we started relocation of the SCSS to the SACLA undulator hall with the addition of one C-band accelerator unit (*i.e.* three units in total) to increase the electron beam energy to ∼500 MeV. After the RF conditioning, we started the commissioning of this machine, which we call the SCSS+, in September 2015, and readily observed the first lasing at *λ* = 33.7 nm (*i.e.* photon energy *h*ν = 36.8 eV) on 7 October 2015. We finished the commissioning of the photon beamline and the construction of the experimental station in June 2016, followed by the first user operation in July 2016.

This paper reports the design and performance of the soft X-ray FEL beamline BL1 at SACLA. The design of the light source, the beamline optics and beam diagnostics, and the experimental station will be described in §2. Characteristics of soft X-ray FEL light are then described in §3 and finally we will show the future perspective in §4.

## 2. Design   

### 2.1. Light source   

**Figure 1:**
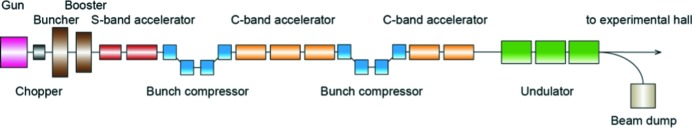
Layout of the SCSS+ accelerator.

The layout of the light source apparatus SCSS+ and the machine parameters are shown in Fig. 1 ▸ and Table 1 ▸, respectively. The electron accelerator and the undulator are wholly located beside the XFEL beamlines BL2 and BL3 in the SACLA undulator hall. The distance from the electron gun to the undulator hall end is 230 m, including a 140 m drift space for future energy upgrade.

The electron gun with a CeB_6_ thermionic cathode launches a microsecond electron pulse with an energy of 500 keV and a peak current of 1 A at a repetition rate of 60 Hz (Togawa *et al.*, 2007 ▸). A central part of 1 ns is cut out from the microsecond pulse using a beam chopper followed by the buncher section that consists of a 238 MHz pre-buncher and a 476 MHz booster (Shintake *et al.*, 2009 ▸). The beam is compressed down to about 10 ps due to velocity modulation by these multi-frequency cavities and bunched after the flight in the drift space. The S-band linac accelerates the bunched beam to 49 MeV while providing a negative energy chirp over the whole bunch to further compress it by the following first bunch compressor (BC1). An energy filter located at the dispersive section of the BC1 removes longitudinal head and tail parts of the bunch; the charge of ∼0.2 nC is sent downstream. The normalized projected emittance of the bunch was measured to be 3 mm mrad by the quadrupole-magnet-scan method at the BC1 exit.

Five C-band accelerator units are used for main beam acceleration (Inagaki *et al.*, 2014 ▸; Sakurai *et al.*, 2017 ▸). Each unit has two 1.8 m accelerating structures, whose maximum energy gain is more than 150 MeV. The first three units accelerate the bunch at an off-crest phase for the compression at the second bunch compressor (BC2); the other two units accelerate the bunch at the on-crest phase to the final beam energy. The final energy can be tuned in the range between 300 and 800 MeV to control FEL wavelengths over a wide range.

**Figure 2:**
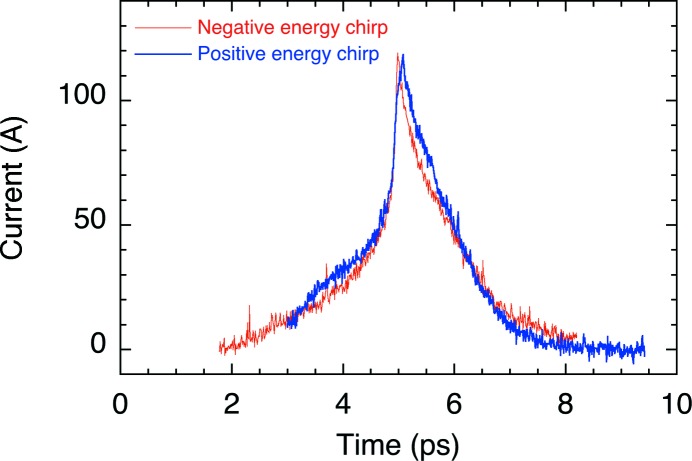
Longitudinal current profiles of the electron beam at the exit of the first bunch compressor measured by means of an RF zero-phasing method. Two profiles obtained by providing positive and negative energy chirps coincide with each other.

The longitudinal bunch structure after the BC1 was measured by an RF zero-phasing method (Wang *et al.*, 1998 ▸). A linear energy chirp was added along the bunch by the zero-crossing phase of the third C-band unit. Then the energy profile, which corresponds to the time structure of the bunch, was analysed at the dispersive section of BC2. The peak current and the width of the bunch were measured to be ∼120 A and 0.8 ps full width at half-maximum (FWHM), respectively, as shown in Fig. 2 ▸. Since there is no diagnostic apparatus such as an RF deflector after the BC2, an exact bunch profile at the undulator section has not been measured so far. Based on the particle tracking simulation using *PARMELA* and *ELEGANT* codes, we estimated that a peak current of ∼300 A and a bunch width of 0.5 ps (FWHM) are achieved after the final compression. The normalized slice emittance of the bunch was analysed to be 1 mm mrad.

Three in-vacuum undulator units, whose magnet design is the same as the SACLA undulator, are used to generate soft X-ray FEL light (Tanaka *et al.*, 2008 ▸). The undulator gap range is from 3.8 to 20 mm; the maximum deflection parameter (*K*-value) is 2.1. The periodic length of the permanent magnet and the total number of periods of the three units are 18 mm and 777, respectively. The FEL radiation wavelength can be varied between 8 and 50 nm by tuning the electron beam energy and the undulator *K*-value. The characteristics of FEL are described in §3.

An electron-beam-based alignment was performed during the undulator commissioning. The electron beam orbits were measured at different beam energies. In order to achieve a dispersion-free condition in the undulator section, the strengths of the steering coils located in front of each undulator unit were determined so that the beam orbit is kept constant while changing the beam energy. After that, the injection position and angle of the beam into the undulator section, the RF phases of the accelerator and the strengths of the focusing magnets were tuned to maximize the FEL pulse energy.

### 2.2. Beamline   

#### 2.2.1. Layout of beamline   

**Figure 3:**
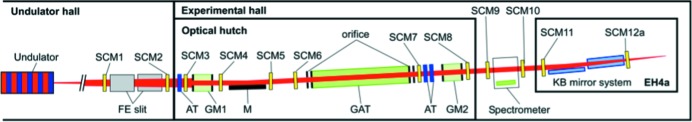
Schematic drawing of BL1 (top view). FE slit: front-end slit; SCM: screen monitor system; AT: foil attenuator; GAT: gas attenuator; M: plane mirror (partial C coating on Si substrate); GM: gas intensity monitor.

Fig. 3 ▸ shows the top view of the photon beamline. It consists of the front-end section in the SACLA undulator hall, the transport channel in the optical hutch (OH) and the experimental station 4a (EH4a) in the SACLA experimental hall. The beamline optics and diagnostics instruments are mainly installed in the OH. Because of the high absorption cross section in the soft X-ray region, the beamline is windowless, and kept at ultra-high vacuum (UHV, <10^−6^ Pa).

#### 2.2.2. Beamline optics   

The optical system of BL1 consists of a plane mirror and gas/foil attenuators. For the plane mirror, we utilize a 400 mm-long Si substrate partially coated with carbon, which provides high reflectivity in a wavelength range longer than the carbon *K*-edge of 4.4 nm at a glancing angle of 1.5°. The plane mirror is installed at a distance from the exit of the last undulator of *L* = 45 m.

The gas and foil attenuators installed in the OH are used to control the FEL pulse energy. The foil attenuators, which consist of Al, Si, Sn, Ti and Zr (see Table 2 ▸ for details), can be combined with the gas attenuator (GAT) at *L* = 50 m to select the fundamental or the higher harmonics. For GAT, a 2.6 m-long chamber is filled with N_2_ with a pressure of up to 100 Pa. To keep the UHV condition through the beam path, differential pumping chambers with 6 mm-diameter orifices are attached to both sides of the GAT.

#### 2.2.3. Beam diagnostics   

Photon diagnostic systems such as photodiodes (SXUV-300C, OptDiode), Ce:YAG screens (Konoshima Chemical) and a spectrometer that combines a variable-line-spacing plane grating (30-002, Shimadzu) with a phosphor-coupled microchannel plate (F2224, Hamamatsu Photonics), are installed in the transport channel of BL1. The spectrometer is calibrated using the absorption spectrum of aluminium *L*_2,3_-edge and the emission spectrum of the He^II^ 2*p* to 1*s* and 3*p* to 1*s* transition.

Since the photon beam parameters can fluctuate shot-by-shot, nondestructive diagnostics are important for analysis of experimental data. For nondestructive diagnostics of the pulse energy, we developed a gas intensity monitor (GM), which works as an ion chamber to detect photoionization of rare gas with an electron multiplier (R2362, Hamamatsu Photonics). In the present study, the chamber of the GM was filled with Ar at a pressure of ∼10^−4^ Pa. The signal intensity was converted to the ion current and finally to the pulse energy through calibration with a calorimeter (Tanaka *et al.*, 2015 ▸). We installed two GMs in the OH: GM1 at *L* = 43 m is used for measuring the pulse energy without attenuators, while GM2 at *L* = 60 m measures the pulse energy after the attenuators. Similar to GAT, we attached differential pumping chambers with 6 mm-diameter orifices to both sides of the GMs. The orifice size was determined so as to transmit more than 95% of the incident pulse energy (4σ width of the beam size) at *λ* = 12.4 nm based on the simulation result using *SIMPLEX* (Tanaka, 2015 ▸).

**Figure 4:**
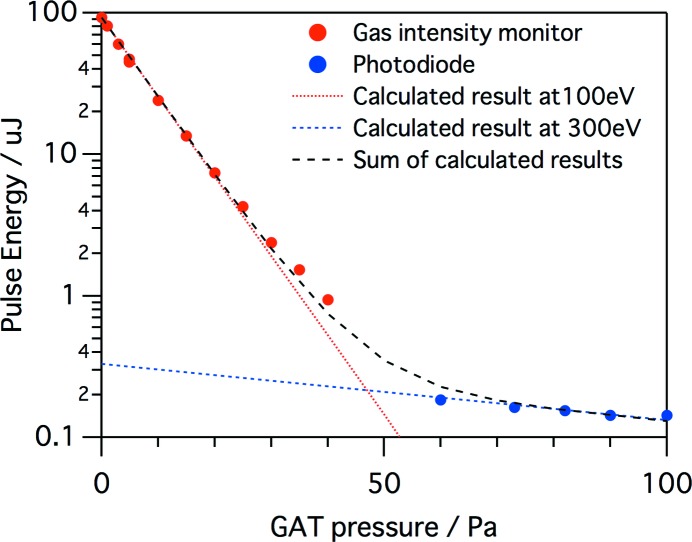
Dependence of FEL pulse energy on N_2_ pressure measured with GMs and the silicon photodiode. The red dashed line shows a calculated result by the Henke’s cross-section data at *λ* = 12.4 nm, while the blue dashed line is that given at *λ* = 4.1 nm. The black dashed line shows the summation of the two calculated results.

By combining these tools, we measured the FEL transmittance at a wavelength *λ* = 12.4 nm while changing the gas pressure of the GAT, as shown in Fig. 4 ▸. We used two GMs for the lower attenuation region from 0 to 40 Pa, while we combined GM1 and the photodiode for the higher attenuation above 60 Pa. The dependence between 0 and 30 Pa agrees with those calculated with the Henke’s cross-section data (Henke *et al.*, 1993 ▸) at *λ* = 12.4 nm, while the small mismatch between 30 and 40 Pa is explained by the contribution of the third-order harmonics of *λ* = 4.1 nm. The measured curve between 60 and 100 Pa is simply reproduced with the calculated result at *λ* = 4.1 nm, which indicates that the third-order harmonics is dominant at the gas attenuator pressure higher than 60 Pa. The contribution of the third harmonics without attenuation is estimated to be ∼0.3%, as seen in Fig. 4 ▸.

### 2.3. Experimental station   

We built one experimental station, called EH4a, at the end of the beamline. A Kirkpatrick–Baez (KB) focusing mirror system and a synchronized optical laser are installed as common-use apparatus. The typical pulse energy at the focus is ∼90% of the pulse energy measured by GM1 in the wavelength range shorter than 30 nm. We do not have an experimental chamber dedicated to the station at the moment, as most users are expected to bring their own UHV chambers.

#### 2.3.1. KB mirror system   

The specification of the KB mirror system at *L* = 85 m is summarized in Table 3 ▸. The sizes of the mirrors are designed to be large enough to accept a beam size of 15 mm, which is sufficiently larger than that restricted by the last orifice (6 mm diameter) of GM2.

**Figure 5:**
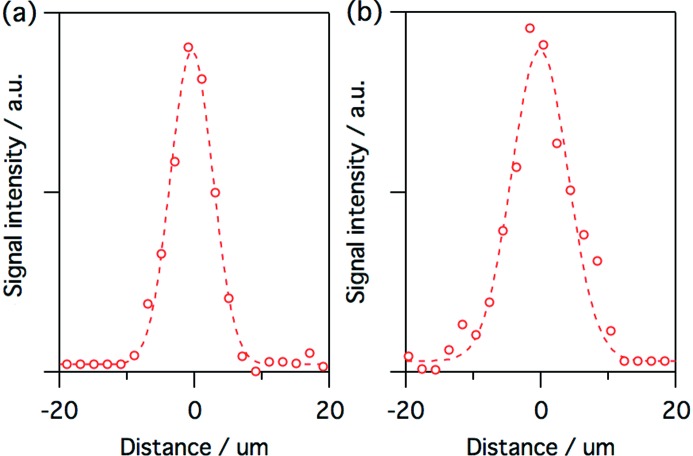
Spatial profiles of the focused beam with the KB mirror in the (*a*) horizontal and (*b*) vertical direction, measured at *λ* = 12.4 nm with the knife-edge scan method. The red circles are measured results, while the dashed lines are Gaussian fits. The knife-edge scan measurement was performed in the vacuum chamber using a gold wire, motorized stages and a silicon photodiode. The soft X-ray FEL pulse energy was reduced using the gas attenuator at an N_2_ pressure of 70 Pa combined with 1.1 µm Zr foil to eliminate the contribution of the higher-order harmonics.

Fig. 5 ▸ shows a focused beam profile at *λ* = 12.4 nm measured using knife-edge scanning of an Au wire (200 µm diameter). The spot size was ∼7.3 µm (FWHM) in the horizontal and ∼10 µm (FWHM) in the vertical. The slight difference between the horizontal and the vertical spot size is due to the difference of the focal lengths. A larger spot size is also available at the defocusing condition. For example, the spot size is enlarged to ∼30 µm in the horizontal (FWHM) and ∼40 µm in the vertical (FWHM) at a location 20 mm upstream of the focal position.

#### 2.3.2. Synchronized optical laser system   

The synchronized Ti:sapphire laser for pump–probe experiments is located in the laser hutch, which is about 13 m away from EH4a. The basic scheme of the laser system and the synchronization system is similar to that employed for BL2 and BL3 (Tono *et al.*, 2013 ▸). The laser system consists of a mode-locked oscillator (Vitara, Coherent Inc.), a chirped pulse amplification system (Legend Elite, Coherent Inc.) and a home-build multi-pass amplifier. This laser system generates a pulse energy of ∼10 mJ with a pulse duration of ∼40 fs at *λ* = 800 nm after the pulse compression performed at the experimental station. The central wavelength in the range 200–2600 nm is available by combining the second, the third and the fourth harmonics of 800 nm, and the optical parametric amplifier (HE-TOPAS Prime, Light Conversion).

The timing of optical laser pulses is synchronized to the master clock of the SACLA accelerator, which is commonly used to drive the SCSS+ accelerator. The cavity length in the oscillator was compensated for synchronization (Synchrolock-AP, Coherent Inc.). For the fine delay scan, a high-precision optical delay stage (custom-made, Kohzu Seiki) with a resolution of 6.7 fs per pulse is installed in the experimental station. For coarse delay scans, a phase shifter (84DgR5B01, CANDOX systems) with a resolution of ∼1 ps can be used.

## 3. Performance   

**Figure 6:**
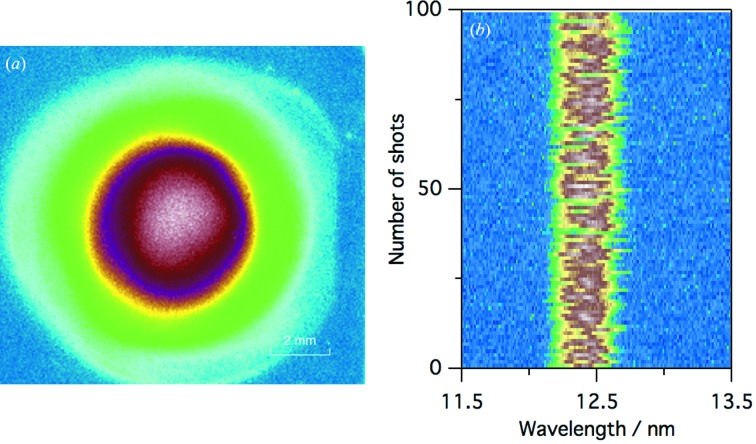
(*a*) Spatial profile of the FEL beam at SCM3 and (*b*) shot-to-shot FEL spectra measured at *λ* = 12.4 nm.

The typical spatial profile at SCM3 (*L* = 42 m) and shot-to-shot spectra of soft X-ray FEL pulses at *λ* = 12.4 nm are shown in Fig. 6 ▸. We observed that the spatial profile was close to a symmetric Gaussian distribution. The spectral resolution was measured to be Δ*λ*/*λ* ≃ 2%.

**Figure 7:**
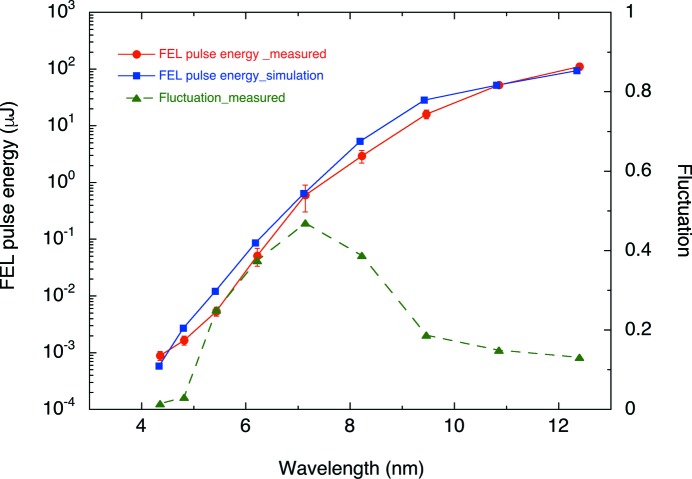
Averaged FEL pulse energy and its shot-by-shot fluctuation as a function of wavelength. The red solid circles are measured pulse energies while the blue circles are simulated ones. The green triangles are the measured fluctuation.

Fig. 7 ▸ shows the averaged FEL pulse energy measured as a function of the undulator *K*-value and radiation wavelength *λ*. The electron beam energy and the bunch charge for this measurement were 780 MeV and 0.23 nC, respectively. The electron beam envelope and the accelerator parameters were optimized at *K* = 2.1. At each *K*-value, the injection beam orbit to the undulator was adjusted to maximize the pulse energy. To cover a high dynamic range across the wide spectral region, we used different detector sets for the pulse energy measurement: a calorimeter and the gas intensity monitor for high pulse energy (>1 µJ) and a silicon photodiode with the gas attenuator at low pulse energy (<1 µJ). Since the iris with 6 mm aperture limits the transverse aperture to the detector, the measured pulse energies are reduced at long wavelengths. To obtain the pulse energies at the undulator, the measured values were corrected by the transmission ratio calculated from the FEL spatial distribution and the iris aperture.

The pulse-energy fluctuation at each *K*-value was also plotted in Fig. 7 ▸. As can be seen from this figure, the fluctuation at the shortest wavelength *λ* = 4.4 nm with *K* = 0.5 was small, the same as the fluctuation of the bunch charge, because only spontaneous radiation was generated in this condition. With increasing *K*-value, the SASE process grew exponentially and the fluctuation reached a maximum of 47% at *λ* = 7.1 nm. The pulse energy continuously increased to 110 µJ while the fluctuation decreased to 13% at *λ* = 12.4 nm; namely, the SASE FEL output was saturated around 10 nm.

Using the measured dependence of the pulse energy on the undulator *K*-value, we estimated the properties of the electron beam combined with *SIMPLEX*. The result is shown in Fig. 7 ▸ together with the experimental data. The beam conditions assumed in the simulation were a Gaussian distribution with a peak current of 300 A, a length of 0.7 ps (FWHM) and a normalized root-mean-squared emittance of 0.5 mm mrad. The simulation based on these assumptions reproduces the experimental data fairly well. The tendency of slight overestimation of the simulated pulse energy could arise from an inaccuracy arising from the assumption of a longitudinal current profile of the electron beam. The actual current profile will be measured directly using an RF deflector or other devices in the future.

Since the SCSS+ accelerator does not have the function to correct second-order nonlinearity of the beam energy chirp, which is generated in the RF acceleration and the bunch compression processes, the peak current is restricted to be a few hundred amperes. In order to increase the FEL pulse energy, a harmonic RF cavity or multipole magnets are planned to be installed for the nonlinear correction.

## 4. Conclusion and perspective   

As the third FEL beamline of SACLA, the soft X-ray FEL beamline BL1 that employs the dedicated accelerator SCSS+ has been successfully developed. The FEL pulse energy reached 110 µJ at λ = 12.4 nm, which agreed with the simulated result. The KB focusing mirror system and the synchronized optical laser system are operated as common experimental infrastructures.

Based on these achievements, user operation of BL1 started in July 2016. In parallel to the operation, upgrades and developments of the beamline continue. For example, we plan to install an arrival-timing monitor between soft X-ray FEL and optical laser pulses (Maltezopoulos *et al.*, 2008 ▸; Harmand *et al.*, 2013 ▸; Katayama *et al.*, 2016 ▸), and a nondestructive spectrometer (Frassetto *et al.*, 2008 ▸; Brenner *et al.*, 2011 ▸) at the experimental station. We will also expand the diameter of the orifices to 10 mm in the transport channel in order to improve the beam profile with increased transmission in long-wavelength regions.

Furthermore, extra space in the SACLA undulator hall is available for installing additional C-band accelerator units and increasing the electron beam energy to ∼1.7 GeV, which enables shortening of the radiation wavelength to below *λ* = 2.6 nm (*h*ν = 470 eV) at *K* = 2.1. Finally, synchronized operation of soft X-ray FEL and hard X-ray FEL pulses can be performed, as the timing of the SCSS+ linac is synchronized with that of the SACLA linac.

## Figures and Tables

**Table 1:** Machine parameters of the SCSS+ accelerator

Parameter	Present value
Electron beam	
Beam energy	800 MeV (maximum)
Bunch charge	0.2–0.3 nC
Peak current	300 A (simulation)
Energy spread (projected)	0.6% (FWHM)
Normalized emittance (projected)	3 mm mrad
Repetition rate	60 Hz
Undulator	
Periodic length	18 mm
Number of undulator modules	3
Total number of period	777
Maximum *K*	2.1
Minimum gap	3.8 mm
Averaged betatron function, β*x*/β*y*	6 m/4 m

**Table 2:** Specification of the foil attenuators

Attenuator	Thickness
Al	0.1, 0.2, 0.3, 0.4, 0.5 µm
Si	0.1, 0.3 µm
Sn	0.1, 0.2, 0.5 µm
Ti	0.1 µm
Zr	0.1, 0.2, 0.5, 1.0, 2.0 µm

**Table 3:** Design of the KB mirrors

	Horizontal	Vertical
Mirror size	600 × 50 × 50 mm	600 × 50 × 50 mm
Coating	Carbon	Carbon
Glancing angle	1.5°	1.5°
Spatial acceptance	15.1 mm	15.1 mm
Focal length	2.65 m	2.00 m
Geometrical spot size	∼3 µm @ *λ* = 10 nm	∼3 µm @ *λ* = 10 nm
